# Hospital resource utilisation from HPV-related diseases in England: a real-world cost analysis

**DOI:** 10.1007/s10198-022-01453-x

**Published:** 2022-05-13

**Authors:** G. Fabiano, A. Marcellusi, F. S. Mennini, P. Sciattella, G. Favato

**Affiliations:** 1 Institute for Leadership and Management in Health, Kingston University, London, UK; 2 Nuffield Department of Orthopaedics, Rheumatology and Musculoskeletal Sciences, University of Oxford, Oxford, UK; 3 Economic Evaluation and HTA (EEHTA)-CEIS, Faculty of Economics, University of Rome “Tor Vergata”, Rome, Italy

**Keywords:** Human papillomavirus, Real world, Resource utilization, Hospital episodes statistics, England, I10, I11, I18

## Abstract

**Background and objective:**

HPV (human papillomavirus) is the virus most often responsible for sexually transmitted infections. The burden of HPV-related diseases on hospital resources represents a major public health problem. The objective of this study was to quantify the lifetime economic burden of HPV-related diseases based on hospital resources from the perspective of National Health Service (NHS) in England.

**Methods:**

Patients’ data were extracted, anonymised and aggregated by NHS digital from Hospital Episode Statistics (HES) database of patients admitted in 2015 and followed for three years. Data on hospitalizations were identified according to the International Classification of Diseases (ICD-10 CM). Health Resource Group (HRG) tariffs and National Reference Costs were used to estimate the hospitalization costs of anal, cervical, genital, oropharyngeal cancers as well as anogenital warts and cervical dysplasia.

**Results:**

A total of 19,296 hospitalized patients were included in the estimation model, (39% was male and 61% female. At admission, the average age was 60 and 50 years old, respectively). Life-time costs per patients diagnosed with oropharyngeal cancer were £16,911 (£17,142 for male and £16,334 for female), penile cancer £12,539, vaginal cancer £12,676, anal cancer £13.773 (£12,590 for male, £14,525 for female). Cervical cancer accounted for £12,721, whereas cervical dysplasia for £3932. Resource used for hospitalized patients with anogenital warts was equal to £872 (£884 and £856 for men and women, respectively). On average, outpatient accounted for 39% of the total lifetime costs.

**Conclusion:**

The results of this study highlight that a substantial amount of resources is utilized for the treatment of HPV-related diseases at hospital level in England. These measures have the potential to inform policy decisions to ensure an optimal use of the NHS resources.

## Introduction

Human papillomavirus (HPV) is the most common sexually transmitted virus and causes a substantial burden of disease in men and women [1]. It is estimated that 75–80% of sexually active individuals will become infected with HPV in their lifetime [2, 3]. The prevalence of HPV remains unacceptably high. In the United Kingdom, there are around 3200 new cervical cancer cases every year (2016–2018) [4]. Moreover, incidence rates for cervical cancer are projected to rise by 43% in the UK between 2014 and 2035, to 17 cases per 100,000 females by 2035 [5]. Genital HPV infections are contracted through unprotected vaginal or anal sexual intercourse and skin-to-skin genital contact. HPV infections that result in oral or upper respiratory malignancies are mostly contracted through unprotected oral sex [6]. Most HPV infections are asymptomatic and resolve within a few months of exposure. However, the infection can progress to form pre-cancerous and cancerous lesions. HPV has been shown to be the cause of several clinically significant conditions.

The availability of vaccines led to the beginning of population wide immunisation programmes in most Western countries. Three vaccines (Cervarix, Gardasil and Gardasil9) are currently approved for the prevention of HPV infection. All of these vaccines are active against the high-risk HPV 16 and 18 strains, while Gardasil^®^ also protects against HPV 6 and 11. Gardasil9 was authorized in the European market in 2015 and protects against nine strains of HPV, including types 6, 11, 16, 18, 31, 33, 45, 52, and 58 [7].

In 2018, following new scientific evidence and advice from an independent panel of experts (Joint Committee on Vaccination and Immunisation—JCVI), the UK Government extended the vaccination programme previously targeted to girls to include adolescent boys and protect them as well against HPV-related diseases. The decision followed that by the Italian Government which, in 2017, was the first of the G9 countries to introduce a universal, recommended and free vaccination for HPV for girls and boys at the age of 12 [8]. Following to the application of new prevention plans, important changes in terms of resource allocation are expected to take place in the next years.

As of today, the costs of treating HPV-related diseases in England are poorly understood. In specific, there is no evidence of studies providing a life-time estimations based on real-world data. Some studies provide snapshots of costs based on horizontal, multiperiod, analyses. For example, a study conducted in England on patients seen for outpatient and inpatient care with pre-cancerous and invasive vaginal and vulval cancer lesions, showed that between 2009/10 and 2014/15, the total cost of pre-cancerous and invasive vaginal and vulval cancer lesions in English secondary care amounted to over £14 million per year [9]. Further, in 2008, the cost of diagnosing and treating genital warts in England was estimated at £17 million per year [10]. Another study based on the UK data showed that the overall costs associated with the detection and treatment of cervical dysplasia, cervical cancer and genital warts accounted for a total of £208 million in 2003 [11].

Despite the above evidences, there has not been any attempt to estimate the lifetime costs of all the HPV-related diseases at hospital level in a single study. Furthermore, many of the published economic evaluations of HPV immunisation use estimations based on the cost of incurred by other countries.

Thus, the objective of this research study is to estimate the lifetime costs of HPV-related diseases in England based on data from Hospital Episodes Statistics (HES). Specifically, the aim of this study is to estimate the current resource utilization for inpatient and outpatient procedures in England on patients diagnosed with anal, cervical, genital, oropharyngeal cancers as well as anogenital warts and cervical dysplasia.

## Methods

A retrospective case series analysis was conducted using patient level data extracted from the Hospital Episode Statistics (HES) database in England between 2015 and 2018. The HES database includes records of all care funded by the English NHS: all admissions, including patients resident outside of England, accident & emergency attendances, outpatient appointments at NHS hospitals in England, and care delivered by treatment centres (including those in the independent sector) funded by the NHS.

### Data collection and analysis

Records were extracted on patients admitted in 2015/16 which was the latest full-year data available from HES at the time of data request. Patient data were collected based on ICD-10 codes which were identified based on advice from expert clinicians as well as previously published literature. Admitted patient care (APC) (inpatient) records were extracted from HES database based on the presence of the following ICD-10 codes: cervical cancer—C53, cervical dysplasia—N87, vaginal and vulvar cancers—C51-2, C57, anal cancer—C21, oropharyngeal cancers C01-2, C04, C06, C09-10, C12-14, C32, C49, penile cancer—C60, C63, anogenital warts—A63. Only patients who did not show any of the required ICD10 diagnoses in the three previous years to hospital admission and did not show any other malignant tumours in the two years prior to their first hospital admission were included. All finished consultant episodes (FCE) and outpatient attendances related to one of the selected ICD-10 codes were extracted from the HES database along with anonymised patient identification numbers to estimate the annual number of hospitalised patients. Each data extract was validated and cleaned prior to delivery by NHS Digital [12, 13]. The number of patients admitted with any of the primary diagnoses related to HPV in each year of the study period was determined by tracing the unique patient identifiers (HESID) assigned to each FCE. A second clean was conducted, checking for patients whose primary diagnoses did not correspond to the correct ICD-10 (231) or were not enrolled in the requested year (2). Mean annual patient numbers were then calculated.

### Costing

The annual management costs for anal, cervical, genital, oropharyngeal cancers as well as anogenital warts and cervical dysplasia were estimated from the health payers’ perspective. NHS funded healthcare providers in England are reimbursed under the Payment by Results (PbR) scheme. The currencies for payment under PbR are healthcare resource groups (HRG).

To derive relevant HRGs for care delivered to patients with any of the HPV-related disease, inpatient FCEs were aggregated into spells of care (from hospital admission to discharge). Core HRGs were then cross-referenced with the National Tariff 2015/18 to estimate the associated spell cost. Costing analyses were performed in Microsoft Excel 2016.

The hospital cost per patient was calculated by first calculating the total HRG costs for each spell recorded in the data across all patients per year. Following this, the average hospital cost per patient was calculated by dividing the total HRG costs each year by the annual number of hospitalised patients in that specific year.

Outpatient costs were estimated by grouping consultations by treatment specialty based on Treatment Function Codes (TFCs) and whether the consultation was the first of a series or a follow-up. Chemotherapy and radiotherapy costs are not included within the National Tariff due to wide regional variations in fees and practice. For such therapies, the HRGs derived from the HES data were cross-referenced with the 2016 National Reference Costs [14].

Average inpatients and outpatients’ costs were then applied to the cohort of patients admitted in 2015 and followed for 3 years. By doing so, we sought to develop cost-per-case estimates that represented the current values of the total direct medical costs accrued from the time of diagnosis to the end of follow-up.

### HPV attribution

Previous studies highlighted a great heterogeneity of data related to the epidemiology of HPV-related diseases. In specific, prevalence rates of HPV DNA and genotype attribution were reported to be very fragmented. To estimate the costs attributable to HPV types 6, 11, 16, 18, 31, 33, 45, 52 and 58, prevalence rates of HPV DNA and genotype attribution per condition were identified through a systematic review of the extant literature.

Specifically, for each condition, we first retrieved the percentage of DNA attribution to exclude all cases that were not a result of HPV infection (Table 1, column a). We then applied prevalence rates from the literature to calculate the individual fractions attributable to genotypes included in HPV9 vaccine (Table 1, column b). These values were multiplied and applied to calculate the fraction of the total costs of HPV-induced malignancies attributable to the nine genotypes by both disease and sex according to the number of estimated incident cases.

**Table 1:** Prevalence of HPV, and HPV9 genotype fractions

Diagnoses	HPV DNA + (%)	Source	HPV 9 attributable fraction (%, min–max)	Source
	a		b	
Cervical cancer	1.00	[15]	0.891 (0.877–0.904)	[15]
Cervical dysplasia	1.00	[16]	0.82 (0.82–0.82)	[7]
Anal cancer	0.87	[15]	0.944 (0.892–0.975)	[15]
Oropharyngeal cancer	0.40	[17]	0.975 (0.937–0.993)	[15]
Vaginal Cancer	0.37	[16]	0.871 (0.788–0.926)	[15]
Penile Cancer	0.54	[16]	0.907 (0.841–0.953)	[15]
Anogenital warts	1.00	[16]	0.9 (0.9–0.9)	[16]

The search was conducted in July 2020 and covered the period 2000–2020. Two independent researchers screened the titles and abstracts. Full-text articles were included if they reported: (1) epidemiological data on the prevalence of HPV DNA, (2) genotype attribution (6, 11, 16, 18, 31, 33, 45, 52 and 58). Only studies derived based on real-world data, archival or population databases, such as national surveys or registries, were included. Due to the heterogeneity of the rates reviewed and the number of diseases involved, no standardization, meta-analyses or adjustments for the pyramidal stratification of the observed population were attempted. Data on cervical dysplasia were not robust, given that cases are not often reported in cancer registries. Therefore, confidence interval could not be extrapolated. The results of our search which was employed to estimate the HPV9 attributable fractions are illustrated in Table 1.

## Results

A total of 19,296 patients diagnosed with anal, cervical, genital, oropharyngeal cancers, anogenital warts or cervical dysplasia were hospitalized in 2015 in England. Overall, 7,604 (39.4%) patients were males, aged on average 61, and 11,692 (60.6%) were females, with an average age of 50 years old. In Table 2 hospitalized patients are reported by age, sex and diagnosis.

**Table 2:** HPV-related hospitalization in England (2015)

	Total (19,297)		Anal cancer (1367)		Anogenital warts (2473)		Cervical cancer (2028)		Cervical dys (4174)		Oropharyngeal (7331)		Penile cancer (543)		Vaginal cancer (1381)	
	*n*	%	*n*	%	*n*	%	*n*	%	*n*	%	*n*	%	*n*	%	*n*	%
Sex																
Males	7604	39.4	593	43.4	1239	50.1					5,229	71.3	543	100.0		
Females	11,692	60.6	773	56.5	1234	49.9	2028	100.0	4174	100.0	2,102	28.7			1381	100.0
Age classes, years																
< 18	151	0.8			101	4.1	3	0.1	4	0.1	39	0.5	3	0.6	1	0.1
18–24	808	4.2			452	18.3	30	1.5	292	7.0	31	0.4	2	0.4	1	0.1
24–44	5465	28.3	102	7.5	1068	43.2	811	40.0	3,072	73.6	325	4.4	28	5.2	59	4.3
45–64	6335	32.8	603	44.1	627	25.4	661	32.6	726	17.4	3198	43.6	176	32.4	344	24.9
65–75	3449	17.9	379	27.7	135	5.5	254	12.5	64	1.5	2104	28.7	154	28.4	359	26.0
75–84	2200	11.4	183	13.4	65	2.6	209	10.3	12	0.3	1227	16.7	116	21.4	388	28.1
85 +	889	4.6	100	7.3	25	1.0	60	3.0			407	5.6	64	11.8	229	16.6

Mean annual costs were calculated for each diagnosis. Life-time costs per patients diagnosed with oropharyngeal cancer were £16,911 (£17,142 for male and £16,334 for female), penile cancer £12,539, vaginal cancer £12,676, anal cancer £13.773 (£12,590 for male, £14,525 for female). Cervical cancer accounted for £12,721, whereas cervical dysplasia for £3932. Resource used for hospitalized patients with anogenital warts was equal to £872 (£884 and £856 for men and women, respectively). On average, outpatient accounted for 39% of the total lifetime costs. In Table 3, we report the inpatient and outpatient cost by disease, sex and hospitalization year.

**Table 3:** Mean annual cost per patient, (2015–2018)

All	Year 1	Year 2	Year 3	Outpatient	Total
Anal cancer	£4344	£1190	£1010	£7229	£13,773
Anogenital warts	£872	£0	£0		£872
Cervical cancer	£5103	£1004	£834	£5779	£12,721
Cervical dysplasia	£1109	£286	£321	£2216	£3932
Oropharyngeal	£6842	£1202	£1071	£7796	£16,911
Penile cancer	£5654	£1193	£1347	£4345	£12,539
Vaginal cancer	£5061	£1158	£1206	£5252	£12,676
Men					
Anal cancer	£4113	£1061	£900	£6516	£12,590
Anogenital warts	£884	£0	£0		£884
Oropharyngeal	£6977	£1272	£1080	£7812	£17,142
Penile cancer	£5654	£1193	£1347	£4345	£12,539
Women					
Anal cancer	£4521	£1288	£1094	£7622	£14,525
Anogenital warts	£856	£0	£0		£856
Cervical cancer	£5103	£1004	£834	£5779	£12,721
Cervical dysplasia	£1109	£286	£321	£2216	£3,932
Oropharyngeal	£6504	£1026	£1047	£7756	£16,334
Vaginal cancer	£5061	£1158	£1206	£5252	£12,676

Year 1 includes costs for enrolment

Further, the total estimate of the annual economic impact on the NHS in England was calculated. As a result, hospitalizations for any of the HPV-related diagnoses accounted, on average, for £ 211.3 million (Table 4).

**Table 4:** Total lifetime costs

Diagnoses	Lifetime direct costs per incident patient	Patients enrolled in 2015	Total lifetime direct costs per diagnosis (mill £)	HPV 9 attributable costs (mill £)^1^
	A	b	a ×b	
Cervical cancer	£12,720.98	2028	£25,798,153	£22,986,155
Cervical dysplasia	£3,931.55	4174	£16,410,283	£13,456,432
Anal cancer (Women)	£14,525.01	773	£11,227,834	£9,231,795
Anal cancer (Men)	£12,589.64	593	£7,465,657	£6,138,442
Oropharyngeal cancer (Women)	£16,334.36	2102	£34,334,824	£13,390,581
Oropharyngeal cancer (Men)	£17,141.59	5229	£89,633,380	£34,957,018
Vaginal Cancer (Women)	£12,675.71	1381	£17,505,155	£5,580,398
Penile Cancer (Men)	£12,539.04	543	£6,808,699	£3,334,765
Anogenital warts (Women)	£855.55	1234	£1,055,753	£950,178
Anogenital warts (Men)	£884.06	1239	£1,095,353	£985,818
Total Cervical Conditions			£42,208,436	£36,442,586
Total Non-Cervical Conditions			£169,126,654	£74,568,994
Total Burden		19,296	£211,335,090	£111,011,581

^1^Calculated as the fraction due to HPV9 on the total cost associated with HPV (DNA +)

The fraction attributable to the nine HPV genotypes was £ 111 million (£ 99.3–£ 121.5) accounting for approximately 53% (47–57%) of the total annual burden of HPV-related diseases in England. Of these, £ 36.4 million (£ 36.1–£ 36.7) was referred to cervical conditions, whereas non-cervical malignancies accounted for £ 74.6 million (£ 63.2–£ 84.7). The fraction of HPV9 genotype costs attributable to men was equal to £ 45.4 million (£ 38–£52.3), while women accounted for €65.6 million (£ 61.3–£ 69.2). This corresponded to 59.1% (57–61%) of total costs attributable to HPV9 infections for women, whereas men accounted for 40.9% (38.3–43%).

## Discussion

The purpose of this study was to estimate the economic burden of HPV-related diseases in England based on data from Hospital Episodes Statistics (HES). Moreover, this study estimated the current resource utilization for inpatient and outpatient procedures on patients diagnosed with anal, cervical, genital, oropharyngeal cancers as well as anogenital warts and cervical dysplasia.

Our study adopted a real-world data approach to estimate lifetime hospitalization risk and costs and an overall estimation of the economic burden associated to HPV infection. Additionally, we included a stratification of the costs by year after the first hospitalization. Therefore, the present study was the first to measure the lifetime economic burden of HPV-related diseases in England considering a longitudinal perspective with newly available administrative data. By estimating the resource consumption attributable to the different diseases in a three-year time horizon, we aimed to estimate the overall economic burden sustained by NHS in England for inpatient and outpatient event associated with HPV-related diseases.

According to the results of this study, the distribution of the patients profile by disease demonstrates the high impact of cervical dysplasia and oropharyngeal condition for adult patients (half of the overall enrolled patients), while cervical cancer and vaginal cancer occur in 18% of the total managed patients. When comparing our results to the available estimates in England, the results of our study confirm the economic burden caused by cervical and vulvar cancers to be major components of the overall direct costs estimations [9]. Also, the estimates related to anal cancers in man and women are consistent with previous analyses, when related to the annual values at year of diagnosis [18]. At the same time, our study confirmed the major economic burden caused by oropharyngeal cancers, which were responsible for 73% of non-cervical conditions. Furthermore, in our study, the economic burden among men represented around half (40.9%) of the total cost associated with the HPV-related diseases analysed, which similar to previously published data in other countries [19–21]. Moreover, this result is in line with the effort to extend the anti-HPV immunization programme to include boys in the National Immunization Plan in the UK.

The present study has several limitations. First, real-world data from administrative archives were only available for inpatients and outpatients, and no assumptions were made regarding the cost of the drugs used for the treatment of these patients. Such partial use of the real costs may have led to the underestimation of the lifetime economic burden due to the lack of information about drugs. However, due to the increased number of outpatient management of these diseases (especially for GW) and the high impact of cancers on the productivity aspects of the diagnosed patients, we can assume that the economic burden estimated in this work represents just one-third of the total impact from the social perspective [19, 22–25].

A second limitation refers to the possible codification problems; therefore some information may be missing and/or be wrongly reported. In this case, our analysis may have missed this information (due to the inclusion criteria), with the risk of underestimating the economic and epidemiological burden of the considered HPV-related diseases. Additionally, not all diseases led to hospitalization and this may be a further source of underestimation. These limitations should be considered in future research; however, in our opinion, they do not undermine the validity of the cost estimates in the present study or their estimated impact on the total economic burden of HPV-related diseases. Future research should address these gaps in epidemiological and cost data to reduce the uncertainty associated with the present estimates.

## Conclusion

This study represents the first economic analysis that estimates the effects of the overall HPV-related disease in England based on real-world data. Similar analyses have investigated only the burden of disease of some of the cancers considered in this study and often referred to the yearly costs sustained by the NHS perspective. This work is one of the first analyses to evaluate the effects of HPV-related disease management based on real-world data.
